# Fertility, Pregnancies and Outcomes Reported by Females with Common Variable Immune Deficiency and Hypogammaglobulinemia: Results from an Internet-Based Survey

**DOI:** 10.1007/s10875-014-0123-3

**Published:** 2015-01-09

**Authors:** Adi V. Gundlapalli, Christopher Scalchunes, Marcia Boyle, Harry R. Hill

**Affiliations:** 1 Departments of Internal Medicine, University of Utah School of Medicine, Salt Lake City, UT 84132 USA; 2 Departments of Pathology, University of Utah School of Medicine, Salt Lake City, UT 84132 USA; 3 Departments of Pediatrics, University of Utah School of Medicine, Salt Lake City, UT 84132 USA; 4 ARUP Laboratories/ARUP Institute of Experimental Pathology, University of Utah School of Medicine, Salt Lake City, UT 84132 USA; 5 VA Salt Lake City Health Care System, Salt Lake City, UT USA; 6 Immune Deficiency Foundation, Towson, MD USA; 7 Immunology 5B114D SOM, University of Utah School of Medicine, Salt Lake City, UT 84132 USA

**Keywords:** CVID, hypogammaglobulinemia, pregnancy, infertility, outcomes

## Abstract

**Background:**

Issues of fertility and pregnancy place an extra burden on females with primary immunodeficiencies. Patients lack reliable information and providers lack guidelines to counsel patients on these anxiety-provoking matters.

**Objective:**

To collate concerns and experiences related to fertility and pregnancy from females with humoral immune deficiencies.

**Methods:**

We conducted an internet-based survey of female patients who self-identified as having a diagnosis of primary humoral immune deficiency.

**Results:**

Responses from 490 women with common variable immune deficiency and 100 with hypogammaglobulinemia were evaluated. The reported fertility measure (% of women who had had a birth) was statistically significantly lower as compared to the general US population (70 % vs. 85 %, *p* < 0.0001) whereas the rates of spontaneous pregnancy loss were comparable. This group reported a total of 966 pregnancies; 72 % resulted in a live birth. A majority of the pregnancies progressed with no incident and with continuation of their IgG replacement therapy; 23 % reported an increase in IgG dosing during pregnancy. Only 15 % of those reporting a first pregnancy indicated that they had been diagnosed with immune deficiency prior to their first pregnancy; these women expressed concern regarding the effect of immune deficiency on their fertility, pregnancy and decision to have children.

**Conclusion:**

With inherent limitations of self-reported responses to surveys, females with humoral immune deficiencies reported relatively good rates of fertility and pregnancies ending in live births. Results of the survey will serve as peer support for patients and inform counseling guidelines for providers.

**Electronic supplementary material:**

The online version of this article (doi:10.1007/s10875-014-0123-3) contains supplementary material, which is available to authorized users.

## Introduction

Primary immunodeficiency disorders (PI), such as common variable immune deficiency (CVID) and hypogammaglobulinemia, affect a small but significant proportion of the population. A 2006 prevalence study performed estimates that approximately 1:1200 individuals in the U.S. are diagnosed with a PI [1]. Although the exact prevalence of CVID is not known, it is estimated that 1 in 25,000 to 1 in 50,000 are affected in the US with equal numbers of males and females [2, 3]. The etiology of these conditions is likely multifaceted and polygenic with complex inheritance patterns [4]. The most common manifestations are a history of infections that may be recurrent, persistent or with unusual organisms. The diagnosis is made with a compatible history, a decrease in serum immunoglobulin G (IgG) levels, and poor or non-responsiveness to a vaccine challenge. Besides low IgG levels, patients also have decreased levels of immunoglobulins such as IgA and/or IgM. The diagnosis of these conditions is often delayed [3, 5] and is made with a compatible history and immunological testing. The management and follow-up of these conditions, along with associated co-morbidities such as chronic and recurrent infections, autoimmune phenomena, malignancy, and long-term sequelae on different organ systems is complex and often requires specific expertise [6, 7]. Immunoglobulin replacement therapy and access to specialty care has resulted in increased numbers of patients experiencing near normalcy with decreased infections, increased life expectancy and accomplishments of significant life events such as education, employment, marriage and motherhood [6–8]. The importance of recognition of the immune deficiency prior to pregnancy and institution of IgG replacement therapy before and during pregnancy cannot be overemphasized as this not only protects the mother from severe infections, but also provides transplacental IgG for the fetus.

Issues of fertility and pregnancy place an extra burden on the diagnosis, management, and long-term sequelae in females with CVID and hypogammaglobulinemia. Primary care providers and specialists frequently encounter such questions from patients and families, with some patients expressing concern with regards to the level of knowledge their providers had [9]. Often heard questions include: Will the immune deficiency affect my fertility? What should I expect during pregnancy? Can I continue my IgG replacement therapy through pregnancy?

Currently, medical providers have to rely on their clinical experience, professional peer support, and limited published literature to counsel and manage patients with CVID and hypogammaglobulinemia with regard to pregnancy. The peer-reviewed scientific literature is limited to experience with pregnancies in small numbers of patients, with few publications noted during the past decade [7, 10–24].

The question of fertility (or infertility) and pregnancy loss in females with CVID and hypogammaglobulinemia is not explicitly addressed in the literature. Anecdotally, there is no specific evidence for infertility in individuals with CVID or clear connections between the two; though secondary effects (chronic sequelae) could lead to infertility [25]. Possible associations mediated through autoimmunity and possibly sensitization to IgA are implied in one review [26] and with IgA deficiency in celiac disease patients in one study [27].

A search of selected consumer health websites on the internet for individuals with immune deficiencies such as IDF Friends [28, 29], Daily Strength [30] [30] and primary immunodeficiency support forum [31] reveals much apprehension, uncertainty, and lack of clarity with regard to immune deficiency, fertility and pregnancy. Informational materials directed to patients are limited and have been noted from non-profit foundation websites such as the Immune Deficiency Foundation [29] and publications such as IG Living [32]. More recently, several Facebook pages dedicated to discussions and support for individuals with CVID have been established for peer support and sharing.

With the objective of documenting the patient perspective and experience, this study was undertaken to elicit responses from adult females with CVID and hypogammaglobulinemia to questions related to their experiences with fertility and pregnancy. We used an internet-based survey to develop a compendium of patient experiences that would inform and improve the counseling of current and future patients with regard to these important issues. The target audience for the results includes patients/families with these conditions and providers who care for them.

## Methods

### Setting

The Immune Deficiency Foundation (IDF) is a non-profit national patient organization dedicated to improving the diagnosis, treatment and quality of life of persons with primary immunodeficiency diseases through advocacy, education and research [29]. Patients and family members are encouraged to register on the website to keep up to date on matters related to immune deficiency disorders and receive mailings from the foundation. The sign-up page explicitly states, “By completing this form, you are also playing a vital role in maintaining the largest database of individuals whose lives are affected by primary immunodeficiency diseases. Your information enables IDF to perform valuable surveys that have been and will continue to be instrumental in advancing the needs of the primary immunodeficiency community.”

### Development and Administration of Internet-Based Survey

A detailed survey was developed with over 50 questions to elicit responses in the following themes: demographics, history of primary immunodeficiency disorders (diagnosis and management) in self and family members, detailed health histories in self and first degree relatives for common and uncommon conditions, perceptions and concerns regarding fertility and pregnancy, impact of having an immune deficiency on the decision to have children, outcomes of attempts to get pregnant, and experiences/outcomes of any pregnancies. (The full survey is available on request from the authors).

A brief introductory email with a clickable link to the URL for the survey website embedded within was sent to email addresses associated with females who voluntarily registered on the IDF website as of March 2012. Two reminder emails were sent to non-respondents during the month of March and April 2012. Questions were sequenced based on responses so as to not force respondents to answer questions that were not relevant to them. Respondents had the option to start, save, and return to complete the survey at their convenience. The survey responses were anonymous and a link to the email addresses was used only to send reminders to non-respondents.

All survey responses were collated and descriptive analyses performed in aggregate using Microsoft Excel, Microsoft Access (Microsoft Corporation, Seattle, Washington) and SPSS Statistical Software (IBM Corporation, Armonk, New York). The percentage of survey respondents that reported a birth (fertility measure) was compared with US national statistics [33] using Chi-square tests. Rates of pregnancy loss (spontaneous abortion or still birth) reported by survey respondents were compared to US national statistics [34] using 1-sample proportions test with continuity correction. The US national statistics for birth rates are calculated as the number of births divided by total population in the given year (s) [35]; thus the numbers of births reported by survey respondents as a group cannot be directly compared to US statistics. The metric reported is the percentage of pregnancies resulting in live births [34]. Terminations of pregnancies reported by survey respondents were compared to US national statistics [36]. Survey responses from those who were diagnosed with PI at the time of their first pregnancy were compared to those who were not diagnosed before their first pregnancy using Fisher exact test.

This study was reviewed and approved by the Institutional Review Board (IRB) of the University of Utah School of Medicine.

## Results

An introductory email with the URL link to the internet-based survey embedded within was sent to 5945 known individual email addresses registered in the IDF database as of March 15, 2012. Of the nearly 1200 who started the survey on-line, 1101 females who were ≥18 years completed the survey. Assuming that 50 % of the registrants in the database are female, the response rate for this survey was 37 % (1101 of 2973). Of these respondents, 490 reported a diagnosis of CVID and 100 reported hypogammaglobulinemia, resulting in a cohort of 590 females whose survey responses were analyzed for this study.

### Demographics and other Characteristics of Survey Respondents

The average age of the 590 female survey respondents was 49 years (median 50 years, range 18–81 years). As shown in Table 1, they had been diagnosed with either CVID or hypogammaglobulinemia an average of 10.4 years prior to this survey. Of the 92 % that indicated that they were on IgG replacement therapy, the average duration of therapy was 9 years (median 7 years, range 1 to 57 years). The most frequent route of IgG replacement therapy was by the intravenous route, administered every 4 weeks and at an average dose of 36 g.

**Table 1:** Characteristics of 590 female survey respondents who self-identified themselves as females with a diagnosis of either common variable immune deficiency or hypogammaglobulinemia

Characteristics	Details
All female	
Self-reported diagnosis, *N* = 590 (%)	
-CVID	490 (83)
-Hypogammaglobulinemia	100 (17)
Age distribution of respondents (years)	Average: 49 Range: 18 to 81 Median: 50
Geographic distribution	All 50 US States, District of Columbia and Puerto Rico
Duration of condition from diagnosis, in years, *N* = 582	Average: 10.4 Range: Less than 1 to 57 Median: 7.1
Duration of IgG replacement therapy, in years, *N* = 545	Average: 9 Range: Less than 1 to 47 Median: 7 years
Modality of IgG replacement therapy ever received, *N* = 590 (%)	
-Intravenous route	485 (82)
-Subcutaneous route	284 (48)
-Intramuscular route	38 (6)
-Intravenous and subcutaneous	226 (38)
-Subcutaneous and intramuscular	23 (4)
-Intravenous and intramuscular	35 (6)
-Intravenous, subcutaneous and intramuscular	22 (4)
-Never treated with IgG	19 (3)
Frequency of IgG replacement therapy	
-Intravenous route, *N* = 267, (%)	
• Every week	11 (4)
• Every 2 weeks	20 (7)
• Every 3 weeks	61 (23)
• Every 4 weeks	161 (61)
• Every 5 weeks	1 (<1)
• Every 6 weeks	12 (4)
-Subcutaneous route, *N* = 232 (%)	
• Daily	1 (<1)
• Twice a week	2 (9)
• Three times a week	1 (4)
• Weekly	198 (85)
• Every 2 weeks	3 (1)
Dose of IgG received as replacement therapy	
-Intravenous route (*N* = 240)	Average in grams
• Every week	34
• Every 2 weeks	32
• Every 3 weeks	40
• Every 4 weeks	36
• Every 5 weeks	25
• Every 6 weeks	28
-Subcutaneous route (*N* = 218)	Average in milliliters
• Daily	10
• Twice a week	40
• Three times a week	38
• Weekly	54
• Every 2 weeks	39

### Medical co-Morbidities in Survey Respondents and First-degree Relatives

The most frequent co-occurring conditions reported by females with CVID or hypogammaglobulinemia were sinusitis, allergies, pneumonia/lung disorders, asthma, and arthritis (Table 2). Cold sores and gallstones were also reported in a significant minority. A number of conditions were reported to be present at the 10 % or greater level in parents, sisters, and brothers; prominent were allergies, sinusitis, pneumonia/lung disorders, and other disorders such as high blood pressure, thyroid and heart disease. Primary immunodeficiencies were reported to occur in very few first-degree relatives (Supplementary Table S1 for details on all conditions reported).

**Table 2:** Medical conditions reported in ≥ 10 % of survey respondents and first-degree relatives, from survey responses of 590 female survey respondents with a diagnosis of common variable immune deficiency (CVID) and hypogammaglobulinemia; (% of total responses to that question). Please see Supplementary Table S1 for responses regarding all conditions

In Survey Respondents (%)	In Mother (%)	In Father (%)	In Sister (%)	In Brother (%)
Sinusitis (76) Allergies (62) Pneumonia/ Lung disorder (55) Asthma (52) Arthritis (43) Thyroid disorder (30) High Blood Pressure (27) Cold Sores (26) Fibromyalgia (24) Intestinal disorders (24) Eczema (17) Urinary Tract Disease (17) Gall Stones (13) Diabetes (12) Stomach/ Duodenal ulcers (11) Cancer (11)	High Blood Pressure (42) Arthritis (37) Allergies (27) Cancer (23) Heart Disease (22) Thyroid disorder (20) Sinusitis (20) Gall Stones (16) Pneumonia/ Lung disorders (15) Diabetes (13) Cold Sores (13) Asthma (12) Intestinal disorders (12)	High Blood Pressure (32) Heart Disease (31) Cancer (23) Allergies (17) Arthritis (17) Diabetes (15) Sinusitis (14) Pneumonia/ Lung disorder (13)	Allergies (18) Sinusitis (12) High Blood Pressure (10) Thyroid disorder (10)	Allergies (14) High Blood Pressure (11)

### Fertility, Pregnancy Loss and live Births Among Survey Respondents

The fertility measure (% of women who had had a birth) reported by CVID and hypogammaglobulinemia patients was statistically significantly lower as compared to the general US population (70 % vs. 85 %, *p* < 0.0001). The rates of spontaneous pregnancy loss (noted as spontaneous abortions by survey respondents) for first and second pregnancies were no greater than the reported national average [34]. Terminations of pregnancies were reported at a calculated rate of 159 per 1000 live births for the first pregnancy (41/1000 and 68/1000 for second and third pregnancies). These are noted to be lower than the US national abortion rate of 228 per 1000 live births reported for 2010 [36].

Nearly three-quarters of all respondents indicated that they did not have difficulty getting pregnant. Similar proportions (72 %) reported that they had ever been pregnant. Of those who had a child with an immune disorder, 60 % indicated that this did not have an impact on their decision to have more children. Overall 72 % of the pregnancies reported by survey respondents resulted in live births.

### Impact of Being Diagnosed With PI on Outcomes and Concerns of Fertility and Childbearing

As it is likely that being pregnant may bias responses to questions regarding outcomes of subsequent pregnancies, we analyzed the impact of being diagnosed with PI at the time of the first pregnancy. Of the 385 women reporting a first pregnancy, 58 reported having had a diagnosis of CVID or hypogammaglobulinemia before the first pregnancy (Table 3). If responses from those who were pregnant during the survey were excluded, there were no differences in outcomes of the first pregnancy between these groups (*p* = 0.8). Rates of infections during the first pregnancy were comparable between the two groups with respondents reporting sinus, ear, urinary and less frequently other infections. Of those whose PI was diagnosed at the time of the first pregnancy, a majority (over 70 %) reported concern regarding their ability to have children, the child developing an immune deficiency, or the pregnancy endangering their health (*p* < 0.001). Those who did not have their PI diagnosed at the time of the first pregnancy expressed little concern. More of those who were diagnosed prior to their first pregnancy indicated concerns regarding PI having an impact on their decision to have or try to have children (*p* < 0.001).

**Table 3:** Results of survey responses for those reporting a first pregnancy: outcomes and responses to questions regarding the impact of being diagnosed with a primary immunodeficiency disorder such as common variable immune deficiency or hypogammaglobulinem

	PI Diagnosed Before First Pregnancy Total *N* = 58 (%)			PI NOT Diagnosed at Time of First Pregnancy Total *N* = 327 (%)		
Outcomes of first pregnancy^1^						
-Pregnant at time of survey -Live Birth -Ectopic pregnancy -Spontaneous abortion/still birth -Terminated pregnancy		5 (9) 40 (69) 0 (0) 7 (12) 5 (9)		1 (<1) 230 (71) 2 (<1) 56 (17) 38 (11)		
Did you experience any infections during this first pregnancy?^1^		Yes: 15 (26) No: 42 (74)		Yes: 71 (22) No: 253 (78)		
	Very concernedN (%)	ConcernedN (%)	Not at all concerned N (%)	Very concernedN (%)	Concerned N (%)	Not at all concernedN (%)
How concerned were you about the ability to have children? ^2^	11 (19)	29 (52)	16 (29)	30 (9)	74 (23)	219 (68)
How concerned were you about your children developing PI? ^2^	22 (38)	31 (53)	5 (9)	12 (4)	26 (8)	278 (88)
How concerned were you about a pregnancy endangering your health? ^2^	10 (17)	36 (62)	12 (21)	15 (5)	57 (18)	247 (77)
Did any of the concerns regarding PI have an impact on your decision to have or try to have children?^2^	Yes: 20 (34 %) No: 38 (66 %)			Yes: 43 (14 %) No: 270 (86 %)		

^1^If responses from those who were pregnant at the time of the survey were excluded, there were no differences in outcomes reported by the two groups (*p* = 0.8)

^2^All responses were significantly different between the two groups (*p* < 0.001)

*PI* Primary immunodeficiency disorder

### Outcomes of Pregnancies and Management of Immune Deficiency During Pregnancy

The 590 survey respondents reported a total of 966 pregnancies with 385 (65 %) reporting at least one pregnancy. The survey allowed responses for up to 10 pregnancies. The majority reported three pregnancies (385 females reported at least one pregnancy, 286 reported two, 153 reported three); with fewer females reporting subsequent pregnancies (number of females reporting fourth through tenth pregnancies respectively: 67, 32, 14, 6, 4, 2, 1). Table 4 shows data for first, second and third pregnancies.

**Table 4:** Responses to questions regarding outcomes of pregnancies and management of common variable immune deficiency and hypogammaglobulinemia during pregnancy

Question	First Pregnancy *N* = 385	Second Pregnancy *N* = 286	Third Pregnancy *N* = 153
How many years ago was this pregnancy?	Average: 24.74 years	Average: 27.42 years	Average: 28.54 years
What was the outcome for this pregnancy? (ALL)	Range: 0 to 63 years	Range: 0 to 62 years	Range: 1 to 59 years
	Median: 24 years	Median: 27 years	Median: 28 years
	N (%)	*N* (%)	N (%)
Live birth	270 (70)	222 (78)	103 (67)
Currently pregnant	6 (2)	2 (<1)	1 (<1)
Ectopic pregnancy	2 (1)	3 (1)	2 (1)
Spontaneous abortion/stillbirth	63 (16)	49 (17)	40 (26)
Terminated pregnancy	43 (11)	9 (3)	7 (5)
Did you go to an immunologist during this pregnancy? (diagnosed patients)	N (%)	N (%)	N (%)
Yes	36 (69)	30 (70)	23 (72)
No	9 (17)	7 (16)	3 (9)
Did not have immunologist	7 (14)	6 (14)	6 (19)
Were you receiving IgG replacement therapy before this pregnancy?	N (%)	N (%)	N (%)
Yes	42 (79)	35 (81)	25 (78)
No	11 (21)	18 (19)	7 (22)
Did you receive IgG replacement therapy during this pregnancy?	N (%)	N (%)	N (%)
Yes	40 (77)	34 (79)	21 (66)
No	12 (23)	9 (21)	11 (34)
Did you continue your IgG replacement therapy during the entire pregnancy?	N (%)	N (%)	N (%)
Yes	38 (95)	38 (95)	21 (100)
No	1 (3)	2 (5)	0 (0)
No answer	1 (3)	0 (0)	0 (0)
While you were pregnant did the number of grams of IgG increase, decrease or stay the same?	N (%)	N (%)	N (%)
Increased	15 (40)	7 (18)	4 (19)
Decreased	1 (3)	0 (0)	0 (0)
Stayed the same	22 (58)	31 (82)	17 (81)
While pregnant did you receive IgG therapy more often, less often or did it stay the same?	N (%)	N (%)	N (%)
More often	6 (16)	3 (8)	4 (19)
Less often	0 (0)	0 (0)	0 (0)
Stayed the same	32 (84)	35 (92)	17 (81)
Did you experience any serious side effect from your IgG therapy during this pregnancy?	N (%)	N (%)	N (%)
Yes	3 (8)	2 (5)	2 (10)
No	35 (92)	35 (95)	19 (90)
Were there any changes to the PI testing you had done during your pregnancy? (Diagnosed only)	N %	N %	N %
Yes	7 (14)	3 (7)	3 (10)
No	45 (86)	38 (93)	28 (90)

Excluding the 10 respondents who indicated they were pregnant during the survey, the overall live birth rate was reported to be 72 %. Nineteen percent (186) reported spontaneous abortion/stillbirth, 7 % (65) reported pregnancy termination (reasons were not reported) and 1 % (9) reported an ectopic pregnancy.

**Fig. 1:**
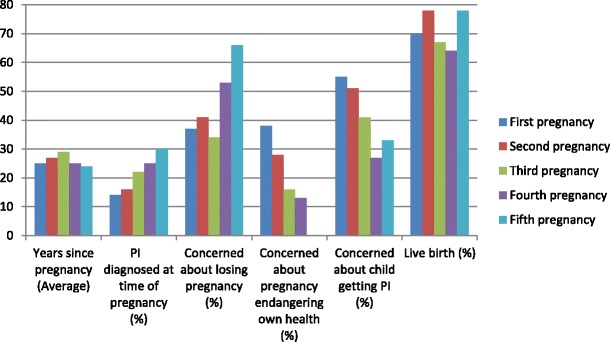
Reported selected concerns and outcomes of first through fifth pregnancies of females with common variable immune deficiency and hypogammaglobulinemia; Results of 939 pregnancies reported for these questions

In reviewing responses for those reporting 1 through 5 pregnancies (Fig. 1), the average years since those pregnancies was between 24 and 29 years ago. Fifteen percent reported a diagnosis of PI prior to their first pregnancy; there was an upward trend in those diagnosed with PI prior to subsequent pregnancies (30 %). There was also an increasing trend in their concern about losing the pregnancy (from 37 to 66 %) from first to subsequent pregnancies. Conversely, concerns regarding the pregnancy endangering their health and their children getting PI were lower with subsequent pregnancies.

Of the 154 pregnancies in which the PI was diagnosed prior to the pregnancy (16 % of all reported pregnancies), the survey respondents indicated that an immunologist had seen them during 106 (69 %) of the pregnancies. The women reported that prior to a majority of these pregnancies (91 %), they had been on IgG replacement therapy. Similarly, IgG replacement therapy was continued in a majority of the pregnancies (86 %) with maintenance of the route of administration (90 %) and frequency (90 %). If a switch occurred, it was from IV route to sub-cutaneous (reasons were not reported). Of those who responded to the question, 23 % indicated that they had an increase in the dose of IgG during their pregnancy (presumably to maintain pace with the weight gain of pregnancy). Nearly all (208, 96 %) of the pregnancies were reported to be uneventful with no serious side effects of IgG therapy. The few patients who experienced side effects were managed by slowing the intravenous IgG infusion rate. There was no change in laboratory testing during 90 % (122 of 136) of the pregnancies. With data available only for the first pregnancy, 77 % of the pregnancies were reported to have been free of serious infections.

With regard to mode of delivery, 20 % of the pregnancies resulting in live births were by C-section (151 of 784 responses). Three females indicated that their immune deficiency influenced the decision to be delivered by C-section. A change to their IgG therapy (dosing and/or frequency) was reported after the completion of 21 % of pregnancies (24 of 114 responses).

## Discussion

Following the principle of “if all else fails, ask the patient,” we present the first descriptive analyses of responses of females with CVID and hypogammaglobulinemia to questions related to fertility and pregnancy. Developing and deploying an internet-based survey directed to self-registered members of a non -profit foundation allowed us to capture responses on a cohort of patients much larger than from studies involving individual clinical immunology practices. With responses from 590 females reporting a total of 966 pregnancies, the self-reported fertility measure of this cohort of females was lower whereas spontaneous pregnancy loss for first and second pregnancies was on par with the general US population. Reported termination rates of pregnancies were lower as compared with US rates. Overall, 72 % of reported pregnancies resulted in a live birth; this compares favorably to that reported for the US in 2008 (65 %) [34].

Overall, the results of this survey should be encouraging to those females and their families who have recently been diagnosed with either CVID or hypogammaglobulinemia. With the caveat that some familial clustering occurs and the genetics of CVID and hypogammaglobulinemia are not completely known, it is likely safe to say that females with these PI could and should consider pregnancy to be a normal part of their lives. Significant concerns regarding fertility and outcomes of pregnancy are noted, especially and predictably, among those who were diagnosed with PI before their first pregnancy. However, overall, females with CVID and hypogammaglobulinemia report that they are able to get pregnant and successfully carry the pregnancy to term.

In considering the survey results and anecdotes from clinical practice, fertility remains a major concern for patients and providers. It is possible that the fertility reported by survey respondents were biased by those who elected not to have children due to their diagnosis of PI. The etiology and treatment of infertility is complex. For patients with PI this likely represents a combination of factors including changes associated with humoral immune deficiency, associated co-morbidities such as autoimmune phenomena, and treatments of those conditions. This area merits further study in terms of epidemiology and mechanisms.

Whereas spontaneous pregnancy loss rates were on par with the general US population, the reported pregnancy termination rates were much lower than US rates. The termination rate was reported to be higher for the first pregnancy as compared to the second and third pregnancies. A multitude of factors including medical, psychological, and social stressors are likely involved in decisions to terminate pregnancies.

It was alarming, though not entirely surprising, to note that only 15 % of patients who reported a first pregnancy indicated that they had been diagnosed with an immune deficiency at the time of their pregnancy. Though this increased during subsequent pregnancies (Fig. 1), this likely poses a risk of unforeseen complications for both mother and infant. This also underscores a continued need for increased awareness and education with regard to immune deficiencies among healthcare providers. Of those who were diagnosed with PI prior to their pregnancy, nearly all continued their IgG replacement therapy with no major side effects. It was also interesting to note that of those who had a diagnosis of immune deficiency, the majority were under the care of a clinical immunologist, which likely accounts for their continuing IgG replacement therapy.

It is important to note that nearly a quarter of those who were on IgG replacement at the time of their pregnancy reported an increase in IgG dose during that pregnancy. Anecdotally, clinical immunologists have considered more frequent testing of serum IgG levels to account for transplacental transfer, increased dosing of IgG during pregnancy to keep up with the weight gain and a ‘booster’ dose prior to delivery [19, 20, 25, 37]. A matching re-adjustment based on weight in the post-partum period has also been noted in clinical practice. Of interest, there were very few changes in IgG frequency or route of administration during and after pregnancy.

The demographics, natural history, routes/doses of IgG replacement therapy, and co-morbidities in the survey respondents and first-degree relatives mirror clinical experience and knowledge recorded in the literature. Some conditions in parents and siblings may reflect age- and gender-related diagnoses such as high blood pressure and heart disease. Diagnoses such as gall stones and cold sores that are anecdotally less noted among females with CVID were reported in a significant minority. It was encouraging to note the low numbers of immune deficiency disorders reported in first-degree relatives. It is important to note that these disorders are often diagnosed later in life and thus may not have been known to the survey respondents (especially in younger relatives).

The overall positive population level results from this cohort with regard to fertility and pregnancy should not detract from the concerns and unfortunate experiences of individual females who have experienced infertility or pregnancy loss while dealing with the complications and sequelae of immune deficiencies. Empathy and understanding are of paramount importance in supporting individuals with chronic diseases through life events.

The self-reported nature of survey responses is a limitation of all such research. Those choosing to participate in the survey may have biased the tone of the responses. There are no data to indicate whether surveys of patients with rare conditions are any more reliable than surveys of patients in general. A perception among IDF database registrants that the survey was meant only for young women who are currently in their child-bearing years may have resulted in lower response rates. Due to differences in demographics and data collection methods, there are inherent challenges in comparing and extrapolating survey responses of a group of self-identified patients with relatively rare conditions to US national statistics.

The survey did not specifically ask about delays in diagnosis that are often noted in immune deficiency patients [3] or issues related to menarche and menopause. Details of infections experienced during pregnancies and reports of neonatal sepsis were not specifically sought in this survey. Clinicians have been concerned about the lack of sufficient antibody to pathogens such as Group B *Streptococci* and *E. coli* in newborns of females with CVID. This merits further study.

Developing formal counseling guidelines based on the results of this survey should be pursued with a broad cross-section of patients, patient advocate groups, providers and professional organizations. Future work should involve follow-up surveys to further elucidate fertility issues, infections and IgG therapy in the peri-natal period including during breastfeeding. There is also a need to understand trends and changes in fertility/pregnancy among the population of females with immune deficiency disorders over time. A long-term prospective data collection study design for those newly diagnosed with immune deficiency and are pregnant would serve to support results of serial cross-sectional surveys. It would also be important to understand these issues as they relate to male patients and male partners of female patients.

## Conclusions

In a first of its kind descriptive analyses of responses to an internet-based survey, 590 females with PI reported on nearly a thousand pregnancies. It was encouraging to note that females with CVID and hypogammaglobulinemia reported relatively good rates of fertility and successfully carrying pregnancies to term (live births). A majority of the females continued their IgG replacement therapy during their pregnancy with no adverse events. Results of the survey will serve as patient peer support. Furthermore, these results should form the basis for improving counseling guidelines for these important and anxiety-provoking topics.

## Electronic supplementary material

Below is the link to the electronic supplementary material.

ESM 1 (DOCX 29 kb)

## Abbreviations

PI: Primary Immune Deficiency Disorder
IgG: Immunoglobulin G
IgA: Immunoglobulin A
IgM: Immunoglobulin M
