# Supplementary material for: Fertility, Pregnancies and Outcomes Reported by Females with Common Variable Immune Deficiency and Hypogammaglobulinemia: Results from an Internet-Based Survey
Source: J Clin Immunol. 2015 Jan 9;35(2):125–34. doi: 10.1007/s10875-014-0123-3 (PMC4352195; doi:10.1007/s10875-014-0123-3)
Supplement: Supplementary file 1 — (DOCX 29 kb) [file 10875_2014_123_MOESM1_ESM.docx]

**Supplementary Table S1.** Medical conditions in survey respondents and first degree relatives, as reported by 590 female survey respondents who are patients with a diagnosis of common variable immune deficiency (CVID) and hypogammaglobulinemia; N (% of total responses to that question). Other diagnoses mentioned by respondents: autoimmune conditions such as Raynaud’s, Sjogren’s, rheumatoid arthritis; inflammatory bowel disease, gastro-esophageal reflux disease, migraines, mitral valve prolapse

|  | **Survey Respondents N (%)** | **Mother N (%)** | **Father N (%)** | **Sister N (%)** | **Brother N (%)** | **Maternal Grand-mother N (%)** | **Maternal Grand-father**  **N (%)** | **Paternal Grand-mother N (%)** | **Paternal Grand-father N (%)** |
| --- | --- | --- | --- | --- | --- | --- | --- | --- | --- |
| PI | 590 (100) | 26 (4) | 15 (3) | 16 (3) | 15 (3) | 7 (1) | 2 (<1) | 2 (<1) | 0 (0) |
| Arthritis | 256 (43) | 217 (37) | 99 (17) | 45 (8) | 35 (6) | 129 (22) | 41 (7) | 34 (6) | 34 (6) |
| Asthma | 309 (52) | 71 (12) | 42 (7) | 49 (8) | 48 (8) | 27 (5) | 10 (2) | 5 (<1) | 6 (1) |
| Allergies | 364 (62) | 157 (27) | 100 (17) | 103 (18) | 82 (14) | 40 (7) | 14 (2) | 14 (2) | 10 (2) |
| Bleeding disorders | 30 (5) | 7 (1) | 10 (2) | 6 (1) | 2 (<1) | 2 (<1) | 4 (<1) | 2 (<1) | 1 (<1) |
| Clotting disorders | 47 (8) | 22 (4) | 11 (2) | 10 (2) | 3 (<1) | 6 (1) | 9(2) | 9 (2) | 5 (<1) |
| Cancer | 63 (11) | 134 (23) | 135 (23) | 41 (7) | 32 (5) | 107 (18) | 86 (15) | 83 (14) | 88 (15) |
| Celiac disease | 32 (5) | 4 (<1) | 1 (<1) | 8 (1) | 5 (<1) | 2 (<1) | 0 (0) | 2 (<1) | 0 (0) |
| Cold Sores | 153 (26) | 74 (13) | 36 (6) | 40 (7) | 17 (3) | 7 (1) | 2 (<1) | 3 (<1) | 2 (<1) |
| Crohn's | 14 (2) | 10 (2) | 7 (1) | 4 (<1) | 0 (0) | 5 (<1) | 1 (<1) | 2 (<1) | 1 (<1) |
| Diabetes | 73 (12) | 78 (13) | 91 (15) | 26 (4) | 34 (6) | 50 (8) | 48 (8) | 56 (10) | 30 (5) |
| Eczema | 102 (17) | 21 (4) | 25 (4) | 26 (4) | 12 (2) | 9 (2) | 5 (<1) | 6 (1) | 5(<1) |
| Fibromyalgia | 144 (24) | 41 (7) | 2 (<1) | 23 (4) | 2 (<1) | 6 (1) | 0 (0) | 3 (<1) | 0 (0) |
| Gall Stones | 79 (13) | 96 (16) | 28 (5) | 29 (5) | 5 (<1) | 39 (7) | 9 (2) | 17 (3) | 4 (<1) |
| Heart Disease | 40 (7) | 131 (22) | 183 (31) | 16 (3) | 36 (6) | 89 (15) | 113 (19) | 72 (12) | 112 (19) |
| Hepatitis | 27 (5) | 17 (3) | 17 (3) | 5 (<1) | 11 (2) | 2 (<1) | 2 (<1) | 7 (1) | 7 (1) |
| High Blood Pressure | 158 (27) | 247 (42) | 190 (32) | 58 (10) | 65 (11) | 95 (16) | 72 (12) | 63 (11) | 56 (10) |
| Intestinal disorder | 139 (24) | 70 (12) | 32 (5) | 35 (6) | 14 (2) | 23 (4) | 12 (2) | 14 (2) | 10 (2) |
| Lupus | 16 (3) | 7 (1) | 1 (<1) | 7 (1) | 1 (<1) | 2 (<1) | 1 (<1) | 0 (0) | 3 (<1) |
| Pneumonia/  Lung disorder | 322 (55) | 87 (15) | 75 (13) | 23 (4) | 23 (4) | 33 (6) | 33 (6) | 23 (4) | 28 (5) |
| Psoriasis | 48 (8) | 11 (2) | 25 (4) | 14 (2) | 4 (<1) | 5 (<1) | 4 (<1) | 6 (<1) | 4 (<1) |
| Sinusitis | 449 (76) | 116 (20) | 83 (14) | 68 (12) | 51 (9) | 28 (5) | 3 (<1) | 9 (2) | 8 (1) |
| Thyroid disorder | 174 (30) | 119 (20) | 36 (6) | 56 (10) | 9 (2) | 37 (6) | 4 (<1) | 15 (3) | 2 (<1) |
| Stomach/Duo-denal ulcers | 66 (11) | 39 (7) | 40 (7) | 11 (2) | 7 (1) | 15 (3) | 11 (2) | 4 (<1) | 4 (<1) |
| Urinary Tract Disease | 99 (17) | 42 (7) | 13 (2) | 24 (4) | 4 (<1) | 6 (1) | 1 (<1) | 5 (<1) | 1 (<1) |
